# Mechanism Study of Thermally Induced Anti-Tumor Drug Loading to Engineered Human Heavy-Chain Ferritin Nanocages Aided by Computational Analysis

**DOI:** 10.3390/bios11110444

**Published:** 2021-11-11

**Authors:** Shuang Yin, Yongdong Liu, Sheng Dai, Bingyang Zhang, Yiran Qu, Yao Zhang, Woo-Seok Choe, Jingxiu Bi

**Affiliations:** 1 School of Chemical Engineering and Advanced Materials, The University of Adelaide, Adelaide, SA 5005, Australia; shuang.yin@adelaide.edu.au (S.Y.); bingyang.zhang@adelaide.edu.au (B.Z.); yiran.qu@adelaide.edu.au (Y.Q.); 2 State Key Laboratory of Biochemistry Engineering, Institute of Process Engineering, Chinese Academy of Sciences, Beijing 100190, China; ydliu@ipe.ac.cn (Y.L.); zhangyao@ipe.ac.cn (Y.Z.); 3 Department of Chemical Engineering, Brunel University London, London UB8 3PH, UK; sheng.dai@brunel.ac.uk; 4 School of Chemical Engineering), Sungkyunkwan University (SKKU), Suwon 16419, Korea; checws@skku.edu

**Keywords:** ferritin, drug delivery, thermally induced drug loading, computational analysis

## Abstract

Diverse drug loading approaches for human heavy-chain ferritin (HFn), a promising drug nanocarrier, have been established. However, anti-tumor drug loading ratio and protein carrier recovery yield are bottlenecks for future clinical application. Mechanisms behind drug loading have not been elaborated. In this work, a thermally induced drug loading approach was introduced to load anti-tumor drug doxorubicin hydrochloride (DOX) into HFn, and 2 functionalized HFns, HFn-PAS-RGDK, and HFn-PAS. Optimal conditions were obtained through orthogonal tests. All 3 HFn-based proteins achieved high protein recovery yield and drug loading ratio. Size exclusion chromatography (SEC) and transmission electron microscopy (TEM) results showed the majority of DOX loaded protein (protein/DOX) remained its nanocage conformation. Computational analysis, molecular docking followed by molecular dynamic (MD) simulation, revealed mechanisms of DOX loading and formation of by-product by investigating non-covalent interactions between DOX with HFn subunit and possible binding modes of DOX and HFn after drug loading. In in vitro tests, DOX in protein/DOX entered tumor cell nucleus and inhibited tumor cell growth.

## 1. Introduction

Mammalian ferritin is a 12 nm symmetrical protein cage consisting of 24 subunits. Each subunit contains a 4-helix bundle (helix A, B, C, and D) and a fifth short helix (helix E). Three N-terminals of subunits gather and form 8 hydrophilic channels in each ferritin shell to allow iron ion penetration [1]. Residues from 4 helices E make another 6 ferritin hydrophobic channels. All 14 channels on each ferritin shell are around 0.3–0.5 nm wide [2]. Ferritin’s unique structure and high biocompatibility have made it a potential drug nanocarrier [3]. Especially, human heavy-chain ferritin (HFn) has shown an intrinsic active tumor targeting ability because it can recognize and bind to human transferrin receptor 1 (TfR1) [4].

Through decades of efforts, research have explored diverse drug loading approaches. Disassembly/reassembly and passive diffusion are 2 mainstream drug loading approaches. A disassembly/reassembly approach involves a dissociation/re-association of HFn assembly induced by pH or 8 M urea. This approach suits drugs either smaller or larger than ferritin channels. However, the disassembly/reassembly process in pH-induced pathway has been criticized. Kim et al. has proven that the process damaged ferritin structure and led to random aggregation of ferritin and drug, in which small aggregates were soluble and huge ones became precipitates [5]. The damage results in 2 problems in drug loading performance: (1) precipitation causes the loss of ferritin and an unsatisfactory protein recovery yield; (2) soluble ferritin-drug aggregates with different sizes can affect drug performance in vitro and in vivo. Condition optimization in drug loading was often required to mitigate these problems. For example, Mehmet et al. and Ruozi et al. critically investigated the pH adjustment course in drug loading and used a stepwise pH adjustment or optimization of final pH to boost protein recovery yield to 55% [6,7]. The 8 M urea-based approach is less frequently used in contrast with the pH-induced one. In two studies, it showed a DOX loading ratio comparable with that of optimized pH-induced approach (around 33 DOX per HFn nanocage), but the protein recovery yield was still undesirable, around 64.8% [8,9].

Passive diffusion approach loads drugs through the hydrophobic or hydrophilic channels on ferritin shell, through incubating ferritin and drugs together under suitable mixing conditions. Different stressors, such as high temperature, additives, and pressure, have been introduced to expand the channels and facilitate drug loading. This approach poses minor effects on ferritin structure and causes relatively low ferritin aggregation and loss compared with the disassembly–reassembly approach. However, the loading efficiency is low [10]. In the study of Yang et al., soybean ferritin was heated with Rutin at 60 °C for 1 h, resulting in a loading ratio of 10.5 Rutin molecules per ferritin [11]. They used chaotropic chemicals, urea, and guanidine chloride, to expand soybean ferritin channels and load molecules in 2 other studies [12,13]. To boost passive loading ratio, Wang et al., have successfully applied high hydrostatic pressure, explored different levels of variables, such as: pressure values, buffer pH, and additives, to finally achieve a 99% of HFn recovery and high DOX loading ratio (32 DOX per HFn nanocage) [14]. However, the high pressurized device is expensive and possesses a number of potential safety risks in operation. Therefore, it is challenging to achieve concomitantly a desirable drug loading ratio and a protein recovery yield in ferritin drug loading process.

In theory, after a drug enters ferritin, it retains in ferritin either by physical entrapment or chemical interaction, or both. For small molecule drugs, such as DOX (molecular weight < 600 Da), chemical interaction dominates. The chemical interaction type and strength are critical to the stability of drug loaded ferritin to prevent drug leakage from ferritin channel. Currently, the chemical interactions between ferritin and DOX have not been investigated in detail. An investigation on these interactions can help understand the drug loading mechanism, interpret the findings in drug loading and lead to an improvement of drug loading performance. In the investigation of protein-ligand binding mechanism, computational tools, molecular docking and molecular dynamic (MD) simulation are significantly regarded and widely used. Molecular docking provides multiple reliable modes of protein-ligand complexes, based on a searching algorithm, whilst MD simulation can assess the validity of these complexes by stability evaluation [15,16]. Shahwan et al. used AutoDock Vina, a molecular docking service, to find the most possible human ferritin (PDB ID: 3AJO)-enzyme inhibitor Donepezil complex, and ran a MD simulation of the complex to assess its stability [17]. These 2 tools are potentially capable of analyzing the chemical interactions between ferritin and drug in loading process.

In this study, a thermally induced passive diffusion was introduced to load DOX to HFn and 2 functionalized HFns, HFn-PAS, and HFn-PAS-RGDK. It is expected to obtain desirable loading results. HFn-PAS was constructed by fusing PAS peptide to HFn C-terminal. HFn-PAS-RGDK was constructed by fusing PAS and RGDK peptide onto the HFn subunit C-terminus. PAS peptide enlarges hydrodynamic volume and RGDK improves inhibition of tumor cell growth through specific affinity with integrin αvβ3/5 and neuropilin-1, which are overexpressed by a wide range of tumor cells [18,19]. Three purified HFn-based proteins were characterized by transmission electron microscopy (TEM) before drug loading. Condition optimization in thermally induced drug loading for HFn and HFn-GFLG-PAS-RGDK were conducted. Size exclusion chromatography (SEC) and TEM were used to detect the structures of proteins after drug loading. DOX loaded proteins (protein/DOX) stability test was performed to check drug leakage profile during storage. For the first time, computational analysis, molecular docking followed by MD simulation, was adopted to analyze chemical interactions contributing to drug loading and aggregation in thermally induced DOX loading process. Finally, in vitro evaluations, intracellular distribution, and cytotoxicity assays, compared 3 HFn-based proteins in vitro performances after thermally induced drug loading.

## 2. Materials and Methods

### 2.1. Materials

Three HFn-based proteins, HFn, HFn-PAS, and HFn-PAS-RGDK were designed as in a previous work [20]. *Escherichia coli* (*E. coli*) BL21 (DE3) (Tiangen Biotech, Beijing, China) was the expression host. MDA-MB-231 cell line was purchased from Cellbank Australia (Sydney, NSW, Australia). Cell culture related reagents were purchased from Thermo Scientific (Massachusetts, MA, USA). All other chemicals of analytical grade except for Doxorubicin hydrochloride (DOX) (Dalian Meilun Biotechnology, Dalian, China), were bought from Chem-Supply (Gillman, SA, Australia). All chromatography columns used in this work were bought from GE healthcare (Waukesha, WI, USA). Millipore purification system (Merck, Melbourne, VIC, Australia) was used throughout the experiments.

### 2.2. Preparation and Characterization of HFn and Functionalized HFns

*E. coli* strains expressing HFn or functionalized HFns were fermented in LB medium at 37 °C and target proteins were expressed by 0.5 mM isopropyl β-d-thiogalactoside (IPTG) 4 h induction. Harvested cell pellets were re-suspended, subjected to ultra-sonication for cell disruption. Lysis supernatants were collected and stored at −20 °C before purification. HFn was purified through the procedure established in a previous work [21]. Harvested *E. coli* lysis supernatants containing HFn-PAS and HFn-PAS-RGDK first underwent 50 °C, pH 5.0, 5 min heat-acidic precipitation to remove host cell proteins, buffer exchange using Hiprep X26/10 G25 desalting column (GE Healthcare, Waukesha, WI, USA), and then pH 7.0 mono Q ion-exchange chromatography (GE Healthcare, Waukesha, WI, USA) for polishing. The 12% reducing SDS-PAGE and TEM were adopted for purity and conformation integrity characterization, respectively. In TEM analysis, a FEI Tecnai G2 Spirit TEM (Eindhoven, NB, The Netherlands) was employed. Operating voltage was 100 kV. Three purified proteins were diluted to 0.1 mg mL^−1^, spread on TEM support grids, air dried, and then negatively stained with 2% uranyl acetate before micrography capture.

### 2.3. Thermally-Induced Passive Loading of DOX into HFn, HFn- PAS-RGDK, and HFn-PAS

DOX was loaded to HFn-based nanocages through thermally induced passive diffusion. Temperature, buffer pH and incubation time are the main factors affecting drug loading. An orthogonal test was designed to optimize thermally induced drug loading condition for HFn and HFn-PAS-RGDK. Variables and levels tested are listed in Table 1.

Initial protein concentration (1 mg mL^−1^) and DOX concentration (0.2 mg mL^−1^) were used in all conditions. Sample buffer was 20 mM phosphate buffer (PB) with 5 mM guanidinium chloride, pH 7.0 or 7.5. After thermal incubation of DOX and HFn-based nanocages, samples were at 1000 rpm 10 min at 4 °C to remove precipitates. Concentrations of the supernatants after centrifugation were measured using Bradford assay (Bio-Rad, Gladesville, NSW, Australia) for calculation of protein recoveries yields. Unloaded DOX was removed using Hitrap G25 desalting column (GE healthcare, Waukesha, WI, USA) and DOX loaded HFn-based protein (protein/DOX) were collected. All protein/DOX peaks then underwent SEC by Superose 6 increase 10/300 GL column (GE Healthcare, Waukesha, WI, USA) to detect if any soluble HFn-DOX aggregates existed.

SEC can separate DOX loaded in HFn-based nanocages (DOX loaded in nanocage) from soluble HFn-DOX aggregates. Peak areas (absorbance at 480 nm) can be used to determine the proportion of DOX loaded in nanocage, using Equation (1). Drug loading ratio, protein recovery yield, and the proportion of DOX loaded in nanocages under various conditions were compared to find the optimal condition. For HFn-PAS, DOX loading was conducted at the optimal loading condition of HFn-PAS-RGDK.

(1) $$\begin{array}{l} \text{Proportion of DOX loaded in nanocage}\;(\%)\\ \;=\frac{Peak\;area\;of\;DOX\;loaded\;in\;nanocage\;}{\mathrm{Peak}\;\mathrm{area}\;\mathrm{of}\;\mathrm{DOX}\;\mathrm{loaded}\;\mathrm{in}\;\mathrm{nanocage}+\mathrm{Peak}\;\mathrm{area}\;\mathrm{of}\;\mathrm{protein}-\mathrm{DOX}\;\mathrm{aggregates}}*100\% \end{array}$$

Drug loading ratio, which is the number of DOX per HFn or functionalized HFn nanocage (N), was determined using Equation (2). C_DOX_ represents DOX concentration in protein/DOX samples collected from Hitrap G25 desalting chromatography. C_nanocage_ represents the concentration of HFn-based proteins in protein/DOX samples. DOX has absorbance at 280 and 480 nm, and protein has absorbance at 280 nm. Therefore, we assume: (1) OD480_nanocage/DOX_ = OD480_DOX_; (2) OD280_nanocage/DOX_ = OD280_DOX_ + OD280_nanocage_. Five standard OD vs. C linear curves were established, including OD480_DOX_ vs. C_DOX_, OD280_DOX_ vs. C_DOX_, OD280_HFn_ vs. C_HFn_, OD280_HFn-PAS_ vs. C_HFn-PAS_, OD280_HFn-PAS-RGDK_ vs. C_HFn-PAS-RGDK_. DOX concentration range for standard curves was 1–40 μg mL^−1^, and concentration range of proteins for standard curves was 0.1–1.2 mg mL^−1^.

(2) $$N=\frac{Number\;of\;DOX}{\mathrm{Number}\;\mathrm{of}\;\mathrm{nanocage}}=\frac{C_{\mathrm{DOX}}•Mw_{nanocage}}{C_{nanocage}•Mw_{DOX}}$$

### 2.4. TEM Characterization of DOX Loaded HFn-Based Proteins and HFn-DOX Aggregate

Three protein/DOX samples under the optimal thermally induced drug loading conditions were analyzed using TEM analysis. A HFn-DOX aggregate sample collected from Superose 6 increase SEC also underwent TEM analysis. Sample treatment and device setting in TEM analysis were the same as in Section 2.2.

### 2.5. Stability of DOX Loaded HFn and Functionalized HFns

After drug loading, the buffer of protein/DOX samples obtained from 50 °C, 6 h, pH 7.5 were exchanged into either phosphate-buffered saline (PBS) pH 7.4. Buffer exchanged samples were placed at 37 and 4 °C. Aliquots were taken from samples at certain time points (0, 2, 4, 8, 24, 72, 120, 168, 336 h) and desalted using Hitrap G25 desalting column (GE Healthcare, USA) to remove leaked DOX, followed by N value calculation.

### 2.6. Computational Study of Interactions of HFn and DOX in Thermally-Induced Drug Loading

Molecular docking and Gromacs MD simulation analysis were used to identify the potential HFn and DOX interactions to explain the formation of HFn/DOX and soluble HFn-DOX aggregates. Molecular docking was performed to analyze the possible poses of HFn subunit and DOX interactions. MD simulation of the docking complexes aimed to find out the most stable HFn subunit-DOX complex structures.

Computational analysis was based on 2 prerequisites: (1) we assume that HFn subunit can be a representative of HFn assembly. This is because the assembly was theoretically 24 repetitions of the subunit. DOX is smaller than HFn channels, which makes it unlikely to simultaneously interact with more than one subunit of the same HFn assembly. (2) The computational analysis focus was on the interactions between DOX and the residues located on HFn assembly outer surface and inner surface. All interactions with interface residues of HFn assembly were ignored.

In molecular docking analysis, PyRx software was used. DOX 3D structure was from Pub Chem and HFn subunit structure file (PDB file) from RCSB PDB (ID: 2FHA). DOX was energy minimized before conducting docking. Top 9 docking HFn-DOX complexes were obtained and saved as PDB files. PDB files from docking results underwent Gromacs MD simulation using Gromacs 2018.

In MD simulation, CHARMM36 force field was used. The HFn-DOX complex structure was solvated in a dodecahedral box of size 460.73 nm^3^ with water molecules and the box was charge neutralized by replacing eight water molecules with 8 Na+ ions. Energy minimization was conducted using the steepest descent integrator for 50,000 steps, until a tolerance of 10 kJ mol^−1^. After this, temperature (NVT) and pressure equilibration (NPT) of the full system were performed at 323 K (approximate 50 °C). Finally, 10 ns 323 K simulation were conducted with 5,000,000 steps and 2 fs each step. Lincs constraint algorithm, Verlet cut-off scheme, Particle Mesh Ewald coulomb type were used in this MD simulation. Root-mean-square deviation (RMSD) and short-range non-bonded interaction energy of each complex in MD simulation were analyzed for the stability assessment. Three-dimensional structures of 9 complexes after MD simulation were saved and the interactions of HFn subunit and DOX within were visualized by Discovery Studio Visualizer. Interactions analyzed include hydrogen bond, salt bridge, and Pi (π) effects. Possible hydrophobic interaction was evaluated by analyzing the residue hydrophobicity in DOX binding area.

### 2.7. In Vitro Anti-Tumor Assessments of DOX Loaded HFn-Based Proteins

MDA-MB-231 is a human breast tumor cell line and has been proven to overexpress human TfR1, neuropilin 1, and integrin αvβ3/5 [22,23]. MDA-MB-231 cells were cultured in L-15 medium with 10% FBS and 1% PS. Intracellular distribution and MTT assay of all 3 protein/DOX were conducted.

MDA-MB-231 cells in exponential growth phase were utilized in intracellular distribution analysis and cytotoxicity assay. Procedures of these two assays were the same as in a previous work using another tumor cell line [20]. A_well_ and cell viability were calculated using the following equations. IC_50_ values of DOX and three protein/DOX were calculated in Origin 9.0 software. Unpaired T test was employed for statistical assessment.

A_well_ = A595 − A630 (3)

Cell viability (%) = (A_well_ − A_blank_)/(A_cell_ − A_blank_) × 100 (%) (4)

## 3. Results

### 3.1. Characterizations of Purified HFn-Based Proteins

**Figure 1:**
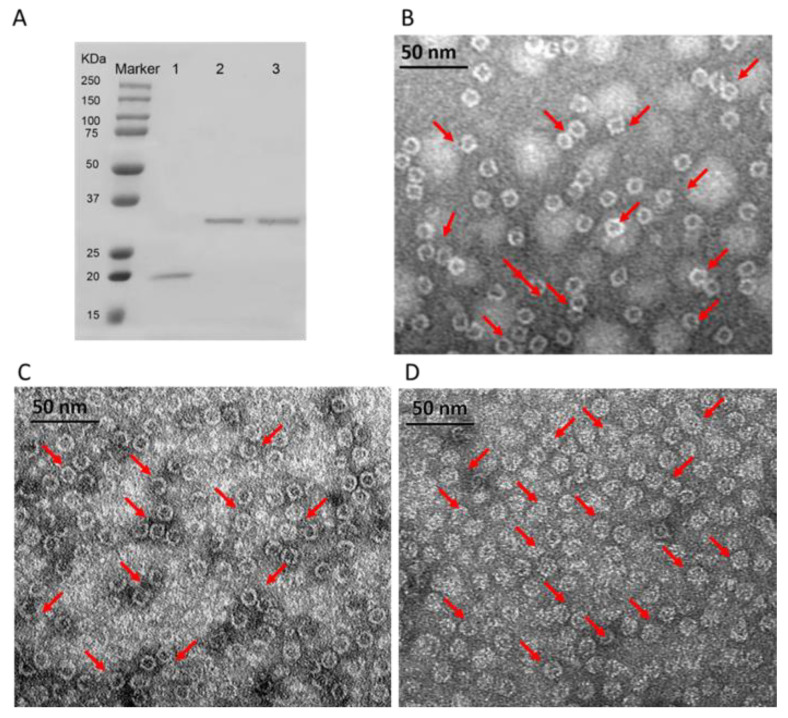
Characterizations of purified HFn-based proteins. (**A**), 12% reducing SDS-PAGE results of purified 3 HFn-based proteins. Lane 1: HFn, 2: HFn-PAS, 3: HFn-PAS-RGDK. (**B**), TEM image of purified HFn. (**C**), TEM image of purified HFn-PAS. (**D**), TEM image of purified HFn-PAS. E, TEM image of purified HFn-PAS-RGDK. Red arrows indicate some spheres.

Figure 1 shows the SDS-PAGE and TEM images of 3 purified HFn-based proteins. In Figure 1A of 12% reducing SDS-PAGE, HFn subunit showed a single band with around 21 kDa. However, 2 functionalized HFns (HFn-PAS and HFn-PAS-RGDK) showed higher apparent molecular weights in SDS-PAGE gel than their theoretical 26 kDa and 26.5 kDa. The discrepancies in molecular weights probably result from PAS peptides, which has the tendency of binding to surrounding water molecules to increase the hydrodynamic radius. Other researchers discovered similar molecular weight increase in PAS modified proteins and PEG-conjugated proteins in SDS-PAGE analysis [18,24].

TEM images in Figure 1B–D demonstrate that both functionalized HFns were assembled hollow spheres, same as HFn. Cages of all proteins were around 12 nm in diameter regardless of functionalization. This is because the inserted functional peptides at the C-terminus did not constitute the ferritin nanocage, while under TEM, the size of the nanocage was visualized.

### 3.2. Optimization of HFn Thermally Induced Passive Loading to Increase Drug Loading

Thermally induced strategy takes advantage of the thermal energy mediated structural perturbation of selective hydrophilic pore areas. Theil and co-workers used Circular Dichroism to analyze the α-helix content change of HFn following heat treatment at different temperatures, and found that a small amount of secondary structure began to transition into random coil when temperature is greater than 45 °C, and it is very likely to take place in pore areas and expand pores [25]. Heating also accelerates Brownian motion of proteins and drug molecules so that greater efficiency could be achieved than in non-heated passive diffusion. In this work, pH 7.0 and 7.5 were chosen to ensure that DOX carries positive charge (DOX pKa 8.3) and HFn inner surface has the opposite charge (HFn pI 4.8). Temperature conditions were selected based on thermal stability of HFn.

**Figure 2:**
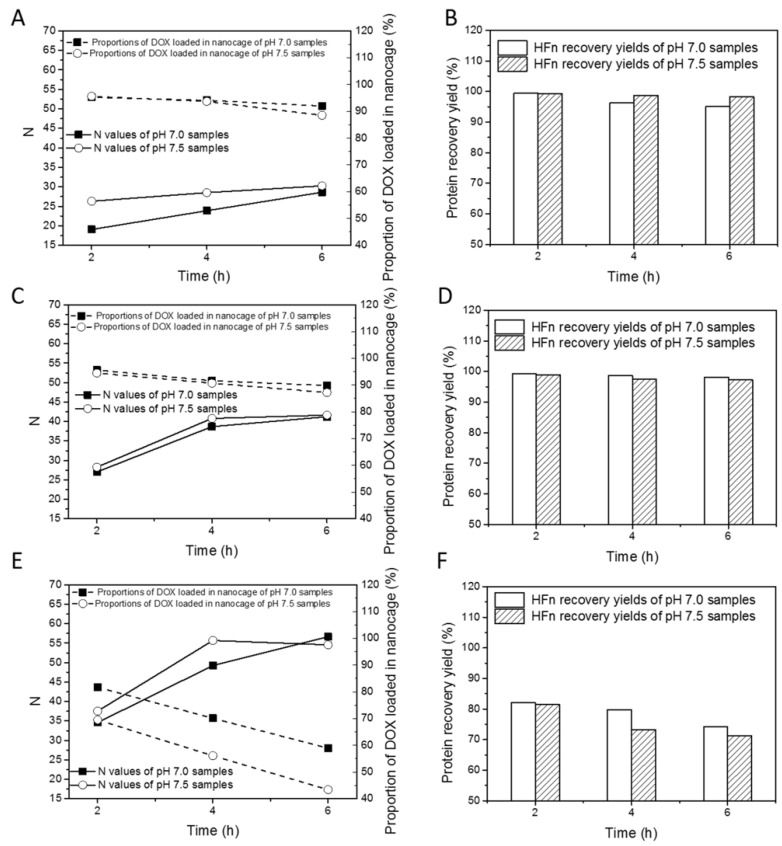
Thermally induced DOX loading results of HFn under different experimental conditions. Loading ratios (Ns) and proportions of DOX loaded in nanocage at 45 °C (**A**), 50 °C (**C**), and 60 °C (**E**). HFn recovery yields at 45 °C (**B**), 50 °C (**D**) and 60 °C (**F**).

Standard curves for determination of drug loading ratio (N) are in Figure S1. Figure 2 summarizes the changes of drug loading ratios, proportions of DOX loaded in nanocage and HFn recovery yields with varying thermal induction time, temperature, and buffer pH. Table S1 lists all drug loading ratios (Ns), proportions of DOX loaded into nanocage and protein recovery yields for HFn. As is shown in Figure 2A,C,E, with the increase in thermal induction duration from 2 to 6 h, N increased at all tested temperatures. At 45 °C, 50 °C, and 60 °C, the highest N was 30.3, 41.6, and 56.7. Ns at pH 7.5 were slightly higher than those at pH 7.0 in most of the time regardless of temperature.

In terms of proportions of DOX loading in nanocage, at 45 and 50 °C, they were at least 85% whilst at 60 °C they were below 85%. At all 3 temperatures, the proportion of DOX loaded into nanocage decreased with the duration of thermal induction. The proportions were largely pH-dependent, and the extent of pH-dependency was subject to temperature, hence they decreased by 0.4–3.5% at 45 °C and 50 °C, and by 10–15% at 60 °C as pH increased from 7.0 to 7.5.

For the HFn recovery yields, in Figure 2B,D,F, at 45 °C and 50 °C, they were above 90%, and at 60 °C, they were mostly below 85%. These results suggest that in the thermally induced drug loading process, DOX loaded in individual HFn nanocages, soluble HFn-DOX aggregates, and HFn-DOX precipitates were simultaneously produced as in previous research using disassembly/reassembly drug loading approaches. At 45 °C, proportion of drug loaded in nanocage and HFn recovery yield decreased slowly, and N increased slowly over time, whilst at 60 °C, proportion of drug loaded in nanocage, HFn recovery yield and N behaved in the opposite manner. These results confirm that 45 °C may not be effective to accelerate drug loading. In addition, at 60 °C, the local structures of HFn nanocages undergo excessive changes, resulting in massive formation of aggregates of HFn with DOX. Considering N, proportion of DOX loaded in nanocage and HFn recovery yield together, 50 °C, pH 7.5, 6 h is the best drug loading condition (N of 41.6, proportion of DOX loaded in nanocage of 87.2% and HFn recovery yield of 97.2%).

### 3.3. Optimization of HFn-PAS-RGDK Thermally Induced Passive Loading

**Figure 3:**
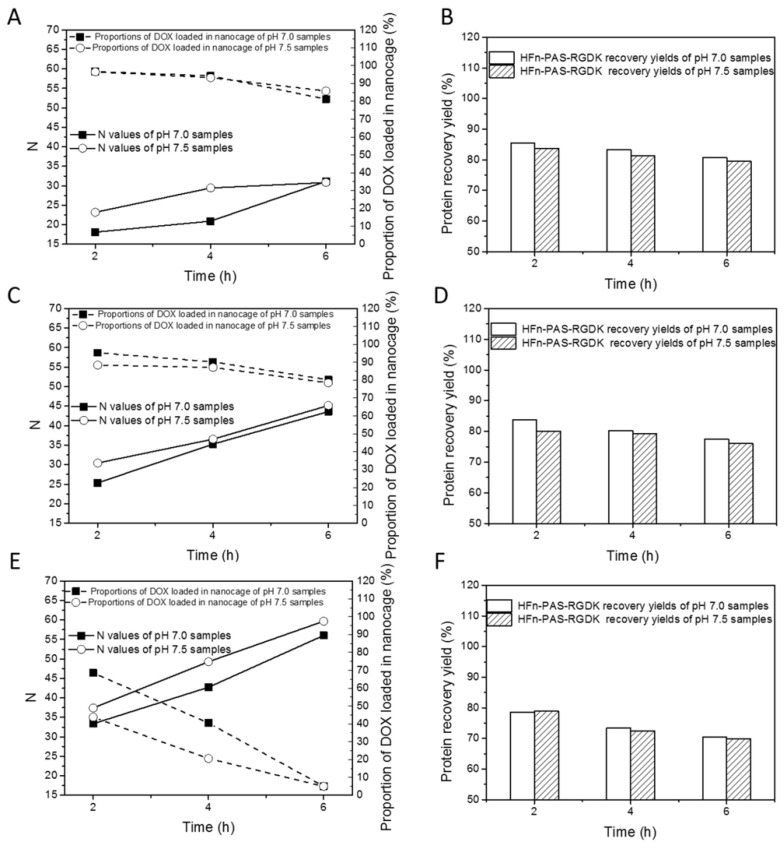
Thermally-induced DOX loading results of HFn-PAS-RGDK under different experimental conditions. Loading ratios (N) and proportions of DOX loaded in nanocage at 45 °C (**A**), 50 °C (**C**), and 60 °C (**E**). HFn-PAS-RGDK recovery yields at 45 °C (**B**), 50 °C (**D**), and 60 °C (**F**).

Figure 3 and Table S2 show the DOX loading optimization results of HFn-PAS-RGDK. The relations between drug loading performance indicators (N, proportion of DOX loaded in nanocage and HFn-PAS-RGDK recovery yield) and experimental variables (induction time, pH and temperature) are similar to those in HFn.

In Figure 3A,C,E, N positively responded to thermal induction duration and temperature. pH 7.5 showed greater Ns than pH 7.0 in most of time. Proportion of DOX loaded in nanocage was negatively related to temperature and incubation time. At 45 °C and 50 °C, proportions of DOX loaded in nanocage were greater than 75%. At 60 °C, they were lower than 70%. As in Figure 3B,D,E, HFn-PAS-RGDK recovery yields were greater than 75% except at 60 °C 4 h and 6 h. The best DOX loading condition was obtained at 50 °C, pH 7.5 and 6 h, with an N of 45.2, proportion of DOX loaded in nanocage of 78.5% and HFn-PAS-RGDK recovery yield of 76.0%. HFn-PAS DOX loading ratio was 38.4, proportion of DOX loaded in nanocage was 73.4% and protein recovery was 75.1% under the same condition.

### 3.4. Conformation of DOX Loaded HFn and Functionalized HFns

**Figure 4:**
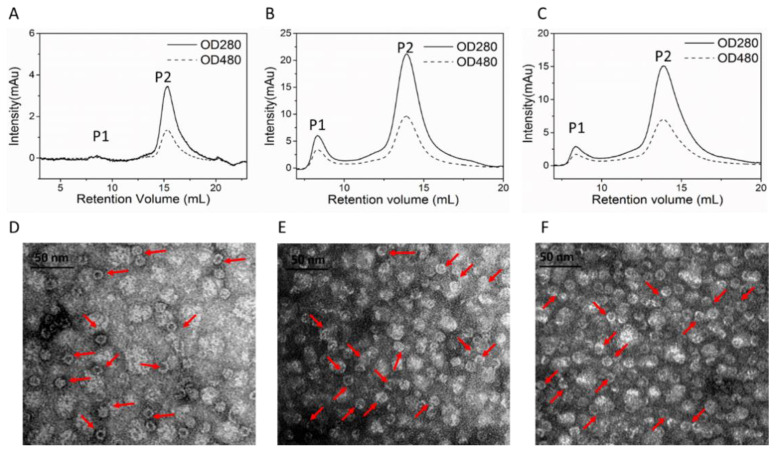
Size-exclusion chromatograms and TEM images of optimal protein/DOX. SEC HFn/DOX (**A**,**D**), HFn-PAS/DOX (**B**,**E**), HFn-PAS-RGDK/DOX (**C**,**F**). Red arrows indicate some spheres.

SEC analysis was performed to prove the success of DOX loading and separate DOX loaded nanocages from HFn-DOX soluble aggregates according to hydrodynamic volume differences. HFn-based proteins have absorbance at 280 nm but not at 480 nm. DOX has absorbance at both wavelengths. Protein/DOX in theory has absorbance at both wavelengths and peak retention time should be similar to HFn-based proteins. Figure 4A–C show the chromatograms of HFn/DOX, HFn-PAS/DOX, and HFn-PAS-RGDK/DOX prepared under the condition of 50 °C, 6 h, pH 7.5. Two peaks, P1 and P2, were observed in all 3 samples. The larger P2 had retention volumes of 13–15 mL in Superose 6 increase column, and absorbance at both 280 and 480 nm. This means it was the DOX loaded HFn-based nanocage. The smaller P1 eluted before P2 was the protein-DOX soluble aggregates, the DOX amount of which accounted for below 27% of the total DOX in the SEC loading samples. The heating process did not affect most of the ferritin nanocage, as are shown in Figure 4D–F. Most of the protein/DOX were hollow spheres. Nanocage sizes were still around 12 nm, the same as before thermally induced passive drug loading process.

### 3.5. DOX Loaded HFn and Functionalized HFns Stability

**Figure 5:**
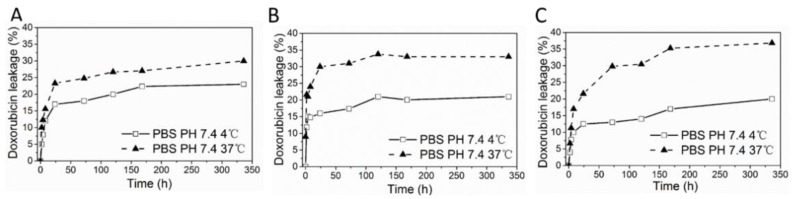
DOX leakage over time at different conditions. (**A**), HFn/DOX. (**B**), HFn-PAS/DOX. (**C**), HFn-PAS-RGDK/DOX.

Protein/DOX stability test was designed to reflect the stability of protein/DOX in storage (4 °C) and blood circulation (37 °C). In storage, drug leakage profiles for all protein/DOX were consistent, where around 20% of loaded drug leaked over 2 weeks (Figure 5). At 37 °C, protein/DOX were less stable in contrast with 4 °C, with around 30% of drug loss detected in HFn/DOX and 35% of drug leaking in other 2 groups for 2 weeks. In all 3 protein/DOX, drug leaked fast during the initial 12 h, then slowed down. Perhaps some of the loaded drugs were just loosely attached or physically trapped inside protein nanocages. Hence, these drugs were more prone to dissociation from protein, while drugs strongly interacted with HFn remained within ferritin. Functionalized HFns showed lower protein/DOX stabilities compared with HFn, which probably result from the insertion of foreign peptides.

### 3.6. Interactions between HFn and DOX in Thermally Induced Drug Loading by Computational Analysis

**Figure 6:**
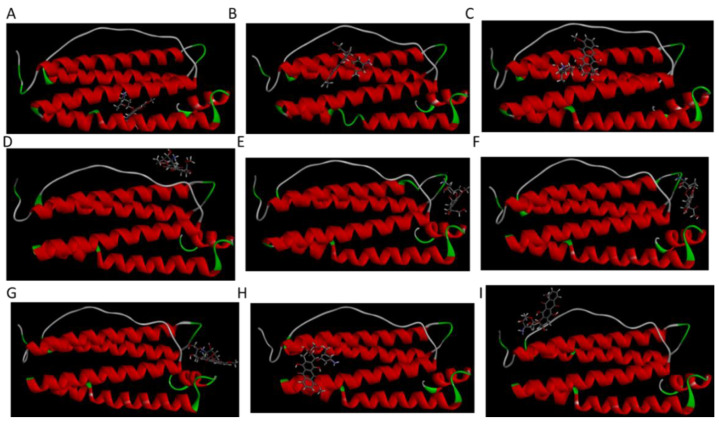
Three-dimensional structures of 9 complexes after 10 ns MD simulation. (**A**–**I**) are complex 1–9.

From molecular docking results, 9 different HFn subunit-DOX complexes were obtained. Complexes underwent 10 ns 50 °C MD simulation for stability assessment. Three-dimensional structures of 9 complexes after simulation are shown in Figure 6. Among them, only in Complex 1, the location DOX binds to was the inner surface in HFn assembly. In the other 8 complexes, DOX bound to areas corresponding to the outer surface in HFn assembly. This implies that Complex 1 is very likely to be the structure of DOX loaded in HFn nanocage, while the interaction ways in the other complexes could form drug loading, soluble HFn-DOX aggregates, and HFn-DOX precipitates in thermally induced drug loading process.

**Figure 7:**
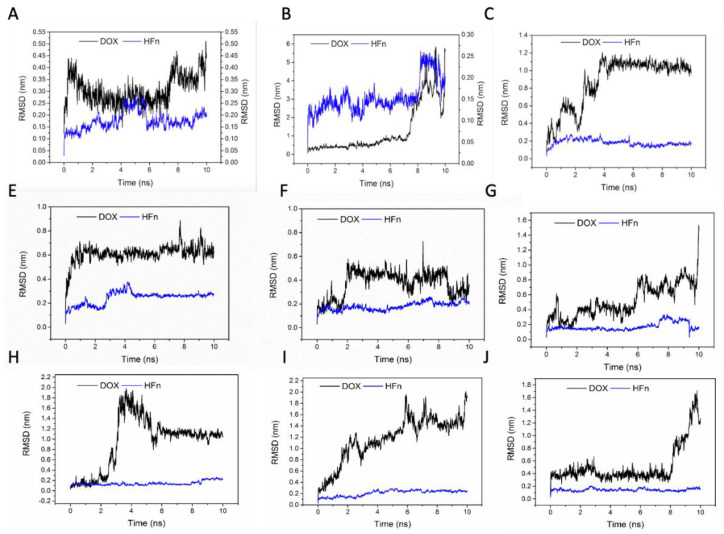
RMSD of HFn subunit and DOX in complexes 1–9 during MD simulation. (**A**–**I**) are complex 1–9.

**Figure 8:**
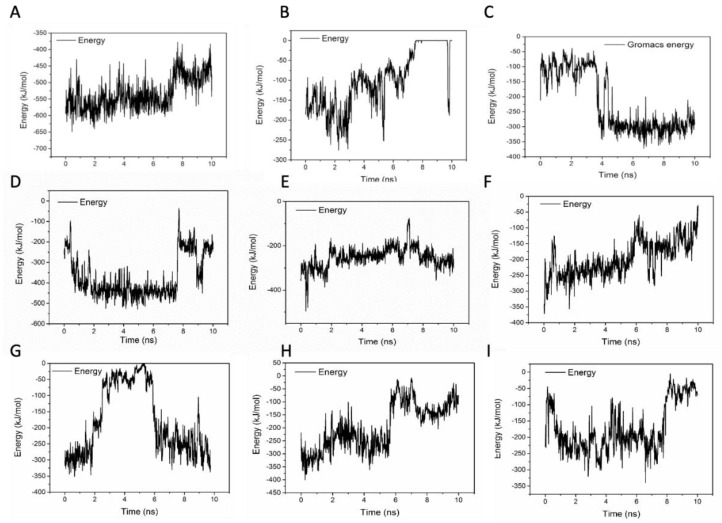
Short-range non-bonded interaction energy of HFn subunit and DOX in complexes 1–9 during MD simulation. (**A**–**I**) are complex 1–9.

To evaluate the stabilities of these structures, the RMSD and the short-range non-bonded interaction energy of HFn subunit and DOX molecule in 9 complexes during simulation were monitored and are presented in Figure 7 and Figure 8. The smaller the RMSD and the lower the energy is, the more stable the complex structure is and the more reliable the complex structure is. The stability orders of structures demonstrated in RMSD and energy are consistent.

Complex 1 was the most stable structure in 50 °C MD simulation, with RMSD lower than 1 nm and energy below −350 kJ mol^−1^. This result is in accordance with the experiment result conducted under 50 °C, that more DOX was being loaded to HFn nanocage than forming soluble aggregates and precipitates. Complex 4 and 5 were the second most stable structures, of which the RMSD were below 1 nm and the energies were below −200 kJ mol^−1^ most of the time. Complex 3 was the third most stable structure. Its RMSD was lower than 1.2 nm. RMSD of Complex 7, 6, 8, 9, and 2 were greater than 1.5 nm and energies of them were above −50 kJ mol^−1^, indicating relatively unstable structures and possibly weak interactions. Based on the stability results, Complex 1, 4, 5, and 3 were the focus in interaction analysis.

Table 2 lists the hydrogen bond, salt bridge and Pi effect interactions between HFn subunit and DOX in Complex 1, 4, 5, and 3 at 10 ns of the simulation. Figure S4 lists the 2D diagrams of 9 complexes of HFn subunit with DOX at 10 ns of the MD simulation. Hydrogen bonds and salt bridges are strong non-covalent bonds, in contrast with van der Waals interaction, such as Pi effects. The more of them in the complex, the more stable the complex structure is. Complex 1 had the most hydrogen bonds and salt bridges.

Regarding the possible hydrophobic interactions between HFn subunit and DOX in Complex 1, 4, 5, and 3, a 5 residue average hydrophobicity was used to reflect the hydrophobicity level of residues in DOX binding area. This is because in a protein, the hydrophobicity of residues can be affected by the nearby residues. Local area hydrophobicity reflects the possibly of hydrophobic interaction better than considering individual residue hydrophobicity. The calculation of 5 residue average hydrophobicity has considered the impact of nearby residues and its value demonstrates how hydrophobic the local area of the residue is. Hydrophobicity values in Table 3 were calculated using Discovery Studio Visualizer. The greater the value is, the more likely it would interact with the hydrophobic DOX molecule. In Complex 1, 4, 5, and 3, there were at least 3 residues at the binding pocket available for hydrophobic interactions with DOX.

According to the computational analysis, in DOX loaded HFn nanocage, DOX was mostly bound to HFn subunit as in Complex 1. Relatively weak binding ways found in Complex 4, 5, and 3 and physically trapped DOX also existed. Therefore, the loading ratio could reach above 24. However, DOX remained in HFn in these weaker ways are more prone to dissociation during storage. Physically trapped DOX probably accounts for the burst release of DOX in the initial 12 h, and the weakly bounded DOX on HFn surface in Complex 4, 5, and 3 would gradually be released, as is shown in Figure 5.

**Figure 9:**
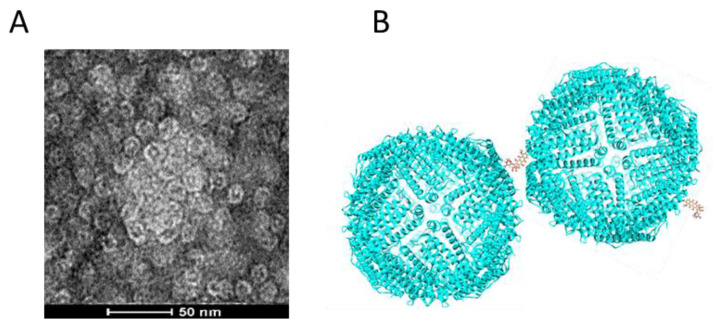
TEM image of DOX loaded in aggregates (**A**) and schematic of conformation of DOX loaded in aggregates (**B**). Cyan part is HFn assembly and brown part is DOX molecule.

Because no aggregates nor precipitates were found in 50 °C 6 h heated HFn, it is reasonable to infer that the interaction of DOX and HFn assembly has led to HFn and DOX aggregation. Small aggregates are still soluble while huge ones turn into HFn-DOX precipitates. TEM image in Figure 9A demonstrates that the HFn in soluble HFn-DOX aggregates were still intact spheres but clumped into a large particle. Interaction ways in Complex 4, 5, and 3 and others, except Complex 1, are theoretically possible to cause aggregation in a way that DOX works as a cross linker (Figure 9B). In each HFn assembly, there are 24 subunits for DOX to bind to, and the HFn-DOX aggregates contain multiple DOX molecules.

### 3.7. Intracellular Distribution and Cytotoxicity of DOX Loaded HFn-Based Proteins

**Figure 10:**
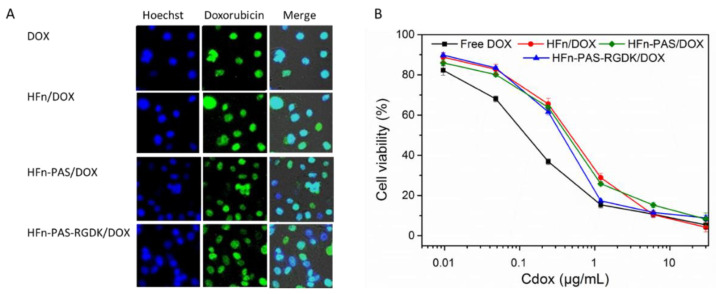
DOX intracelluar distribution photos and cytoxocity comparison of protein/DOX and DOX. (**A**), DOX distribution inside cells shown under cell imager. Blue dots show the locations of cell nucleus. Green dots represent the accumulation of DOX molecules. The light cyan dots in merge photos indicate the DOX molecules accumulated at cell nucleus. (**B**), Cytotoxocity effects of protein/DOX and DOX on MDA-MB-231 cells.

DOX has been proven to be able to diffuse into cell nucleus and disrupt cell division [26]. However, in theory, the DOX loaded on HFn and functionalized HFns need to be released from protein prior to exerting its function. Intracellular distribution test aimed to check whether the release of DOX form protein/DOX occurred. Cell nucleus locations were visualized as blue dots under cell imager after Hoechst 33,258 staining (Figure 10A). Due to the intrinsic fluorescence of DOX molecules, under the excitation of 480 nm light, DOX molecule accumulation could be observed as green dots. In the merged images, the color of dots in all four groups changed to light cyan, indicating that DOX molecules loaded on proteins through thermally induced loading approach were released and accumulated inside cell nucleus.

MTT assay was designed to compare the inhibition effects of DOX loaded on HFn, two functionalized HFns, and free DOX. Figure 10B shows the cell viabilities at different concentrations of equivalent DOX. Table 4 lists the IC_50_ values of all four groups. Free DOX possessed the lowest IC_50_. However, it does not indicate free DOX has the greatest anti-proliferation effect. This is because in in vitro assays, the direct incubation of free DOX with cells has maximized the internalization efficiency of free DOX. On the contrary, the uptake efficiency of DOX in in vivo tests and in real practice would be greatly hampered by the blood circulation and metabolism system. Except for free DOX, DOX loaded on HFn-PAS-RGDK had the lowest IC_50_ value, followed byHFn-PAS/DOX. HFn/DOX had the greatest IC_50_.

No statistical significance was found between anti-proliferation abilities of HFn-PAS-RGDK/DOX group and free DOX group (*p* > 0.05). Anti-proliferation effect of HFn-PAS-RGDK/DOX was significantly higher than the other two HFn-based protein/DOX groups (*p* < 0.05). This is because of the tumor targeting ability of the inserted RGDK in HFn-PAS-RGDK.

## 4. Discussion

In this study, the thermally induced passive diffusion approach succeeded in loading DOX into HFn and 2 functionalized HFns. 50 °C, pH 7.5 and 6 h was found to be the optimal condition for HFn and functionalized HFns. Temperature and incubation time showed a great impact on DOX loading performance. Although HFn and DOX have outstanding thermal stabilities, in the thermally induced drug loading process, both drug loading and irreversible HFn-DOX aggregation occurred under all selected conditions. With the same incubation time, as incubation temperature increased, N value increased whilst the proportion of DOX loaded in nanocage declined. At the same incubation temperature, N value increased and the proportion of DOX loaded in nanocage decreased over incubation time, especially at 60 °C.

Table 5 compares HFn drug loading performance of this work with some previously published studies. In this study, N of HFn (41.6) is greater than previously studies, which adopted 8 M urea or optimized stepwise pH-induced disassembly-reassembly approaches. Recovery yield of HFn in this study, 97.2%, is similar to high hydrostatic pressure passive diffusion approach (99%) and significantly greater than the pH-induced (25%, 55%) or 8 M urea-based approach (64.8%). Disassembly/reassembly approach has been questioned to be not fully reversible because 2 holes were detected by synchrotron small-angle X-ray scattering, and the authors argued that this structural damage may result in protein loss and aggregation in the drug loading process [5]. To the contrary, at 50 °C, HFn nanocage remains intact throughout the thermally induced drug loading process, which involves less structural changes.

Compared with HFn, under most experimental conditions, especially at 50 °C and 60 °C, the functionalized HFn, HFn-PAS-RGDK, had relatively low protein recovery yields and low proportions of DOX loaded in nanocage. HFn-PAS also demonstrated reduced proportions of DOX loaded in nanocage and protein recovery yields. Two functionalized HFns were more prone to aggregation in the heating process, suggesting slightly decreased thermal stabilities. This could be ascribed to the ‘flip to flop’ phenomenon in functionalized HFns, where E-helix with inserted functional peptide are extruded outside HFn nanocage, as was discovered in our previous work [20]. Hydrophobic interactions of 4 helices E around each hydrophobic channel in natural ‘flip’ HFn have been proven to contribute to HFn stability [29,30]. The turnover of E-helix has hampered helices E interactions and thus declined thermal stability.

Combining the results from molecular docking, MD simulation, and experiments, hydrogen bond and salt bridges between DOX and HFn residues in Complex 1 probably account for most of the loading of DOX. Physical entrapment of DOX in HFn assembly and interactions in other complexes may also contribute but they suffer from a rapid DOX leakage during storage, as shown in Figure 5. In the process of thermally induced DOX loading, DOX may undergo unexpected interactions with multiple HFn assemblies through hydrogen bonds and salt bridges to form HFn-DOX aggregates (Figure 9B).

In vitro tests demonstrate that DOX loaded through thermally induced passive diffusion could exert anti-cancer function as free DOX.

## 5. Conclusions

A thermally induced drug loading approach has improved DOX loading ratios and protein recovery yields of HFn and functionalized HFns, HFn-PAS and HFn-PAS-RGDK. This mild and efficient strategy can become an alternative to produce HFn-based nanocages with various drugs. According to molecular docking and MD simulation analysis, hydrogen bond, salt bridges and other non-covalent interactions between HFn and DOX molecules contribute to DOX loading and by-product formation. The combination of molecular docking and MD simulation analyses can be a useful tool to shed light on ferritin drug loading mechanism. In vitro tests show that thermally-induced DOX loaded HFn-based proteins can exert tumor inhibition of DOX. RGDK has promoted DOX internalization to tumor cells and enhanced HFn anti-tumor efficacy.

## Figures and Tables

**Table 1:** Levels of variables used in the orthogonal tests for optimization of thermally induced DOX loading to HFn and HFn-PAS-RGDK.

Variables	Level
Temperature	45, 50, 60 °C
Phosphate buffer pH	7.0, 7.5
Incubation time	2, 4, 6 h

**Table 2:** Interactions between HFn subunit and DOX in complexes.

Complex	Residues Forming Hydrogen Bond with DOX	Residues Forming Salt Bridge/Attractive Charge with DOX	Residues Have Pi Effects with DOX
1	GLN58, GLU62, HIS65, GLN141	GLU27, GLU62 (2) ^1^, GLU107 (2) ^1^	HIS57 (Pi-Pi stacked), TYR54 (Pi-alkyl)
4	ARG43, ASP91	ASP91	TYR39 (Pi-Pi stacked), TYR39 and PRO88 (Pi-alkyl)
5	TYR40, ASP45, GLU94, GLU167	ASP45	/
3	/	/	TYR29 (Pi-Pi T shaped), LEU26 (Pi-alkyl)

^1^ Numbers in the brackets after the residue are the number of the interactions involving the residue.

**Table 3:** Five residue average hydrophobicity of residues in DOX binding area in complex 1, 4, 5, and 3.

Complex	5 Residue Average Hydrophobicity Values of Hydrophobic Residues in DOX Binding Pocket
1	TYR34 (0.92)^1^, TYR54 (0.64) ^1^, LYS143 (0.62) ^1^, ALA144 (0.54) ^1^, GLU147 (0.1) ^1^.
4	TYR32 (0.52) ^1^, SER36 (0.56) ^1^, TYR39 (0.26) ^1^.
5	VAL46 (0.56) ^1^, ALA47 (0.48) ^1^, LEU48 (0.48) ^1^.
3	LEU26 (1.02) ^1^, GLN83 (0.82) ^1^, GLN112 (0.04) ^1^, GLU116 (0.94) ^1^.

^1^ Numbers in the brackets after the residues are the 5 residue average hydrophobicity.

**Table 4:** IC50 values of all groups.

Group	IC_50_ (μg mL^−1^)
DOX	0.15 ± 0.01
HFn/DOX	0.57 ± 0.02
HFn-PAS/DOX	0.46 ± 0.01
HFn-PAS-RGDK/DOX	0.34 ± 0.01

**Table 5:** Comparison on DOX loading to HFn in this work and previous studies.

Protein	Loading Approach	N	Protein Recovery (%)	Reference
HFn	Thermal induction	41.6	97.2	This study
Horse spleen ferritin	Step-wise pH induction	28	55 ± 7	[6]
HFn	pH induction	29 ± 3	40 ± 4	[18]
Equine spleen ferritin	pH induction	22 ± 1	25	[27]
HFn	pH induction	28.3	/	[28]
Ferritin	Urea-based	32.5	64.8	[8]
HFn	Urea-based	33	/	[9]
HFn	High hydrostatic pressure	32 ± 2	99	[14]

‘/’ means no data.

## Data Availability

The data presented in this study are available on request from the corresponding author.

## Supplementary Materials

The following are available online at https://www.mdpi.com/article/10.3390/bios11110444/s1, Figure S1: Standard linear curves of correlations between drug or HFn-based protein nanocages concentrations and optical densities. Table S1: Loading ratios (Ns), proportions of DOX loaded in nanocage and protein recovery percentages in HFn thermally induced drug loading optimization. Table S2: Loading ratios (Ns), proportions of DOX loaded in nanocage and protein recovery percentages in HFn-PAS-RGDK thermally induced drug loading optimization. Figure S2: Size exclusion chromatograms of all HFn/DOX samples under 18 conditions in thermally induced drug loading optimization. Figure S3: Size exclusion chromatograms of all HFn-GFLG-PAS-RGDK/DOX samples under 18 conditions in thermally induced drug loading optimization. Figure S4: Hydrogen bond, salt bridge, and Pi effect interactions between HFn subunit and DOX in Complex 1–9 after 10 ns MD simulation.
