# Supplementary material for: Mechanism Study of Thermally Induced Anti-Tumor Drug Loading to Engineered Human Heavy-Chain Ferritin Nanocages Aided by Computational Analysis
Source: Biosensors (Basel). 2021 Nov 11;11(11):444. doi: 10.3390/bios11110444 (PMC8615661; doi:10.3390/bios11110444)
Supplement: Supplementary file 1 [file biosensors-11-00444-s001.zip › biosensors-1440465-supplementary.pdf]

biosensors MDPI

Supplementary

Mechanism study of thermally induced anti-tumor drug loading to engineered human heavy-chain ferritin nanocages aided by computational analysis

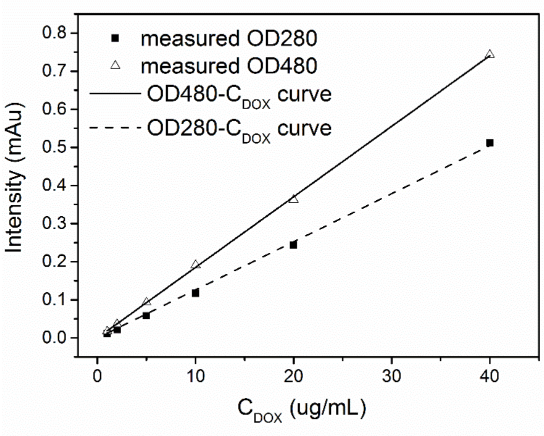

(A)

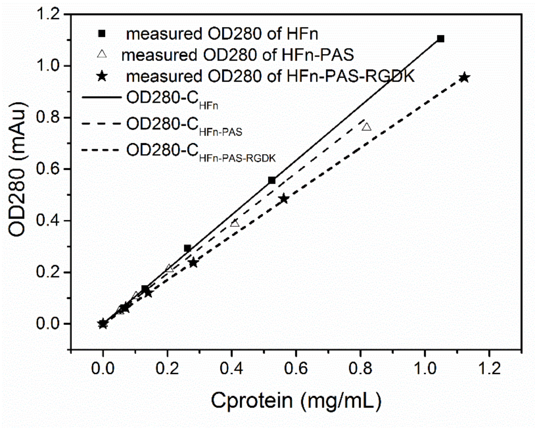

(B)

Figure S1. Standard linear curves of correlations between drug or HFn-based protein nanocages concentrations and optical densities. (A) OD480-CDOX and OD280-CDOX correlation curves. OD280=0.0126 CDOX, R2 = 0.999, OD480 = 0.0185 CDOX, R2 = 0.999 (B) OD280-Cnanocage correlation curves; HFn, OD280= 1.0561 C, R2 = 0.999; HFn-PAS, OD280= 0.9258 C, R2 = 0.998; HFn-PAS-RGDK, OD280= 0.8529 C, R2 = 0.999

Table S1. Loading ratios (Ns), proportions of DOX loaded in nanocage and protein recovery percentages in HFn thermally induced drug loading optimization.

Temperature	Thermal induction duration	Buffer pH	N	Proportion of DOX loaded in nanocage (%)	Protein recovery (%)
45 ºC	2 h	7.0	19.06	95.2	99.5
		7.5	26.30	95.6	99.3
	4 h	7.0	23.88	94.1	96.3
		7.5	28.51	93.7	98.7

	6 h	7.0	28.62	92.0	95.1
		7.5	30.26	88.5	98.2
50 ºC	2 h	7.0	27.04	95.6	99.2
		7.5	28.28	94.5	98.9
	4 h	7.0	38.69	91.6	98.7
		7.5	40.80	90.7	97.5
	6 h	7.0	41.24	89.8	98.1
		7.5	41.64	87.2	97.2
60 ºC	2 h	7.0	34.60	81.7	82.1
		7.5	37.53	69.5	81.5
	4 h	7.0	49.25	70.1	79.8
		7.5	55.69	56.1	73.3
	6 h	7.0	56.67	58.9	74.2
		7.5	54.56	43.4	71.2

Table S2. Loading ratios (Ns), proportions of DOX loaded in nanocage and protein recovery percentages in HFn-PAS-RGDK thermally induced drug loading optimization.

Temperature	Thermal induction duration	Buffer pH	N	Proportion of DOX loaded in nanocage (%)	Protein recovery (%)
45 ºC	2 h	7.0	18.10	96.7	85.5
		7.5	23.20	96.5	83.7
	4 h	7.0	20.92	94.3	83.3
		7.5	29.43	93.3	81.3
	6 h	7.0	31.13	81.3	80.7
		7.5	30.84	85.8	79.5
50 ºC	2 h	7.0	25.35	95.3	83.7
		7.5	30.44	88.4	80.0
	4 h	7.0	35.29	90.2	80.23
		7.5	36.54	87.1	79.3
	6 h	7.0	43.60	80.2	77.4
		7.5	45.16	78.5	76.0
60 ºC	2 h	7.0	33.37	68.5	78.5
		7.5	37.41	43.7	78.9
	4 h	7.0	42.73	40.5	73.4
		7.5	49.28	20.6	72.5
	6 h	7.0	56.03	5.1	70.4
		7.5	59.68	5.0	69.9

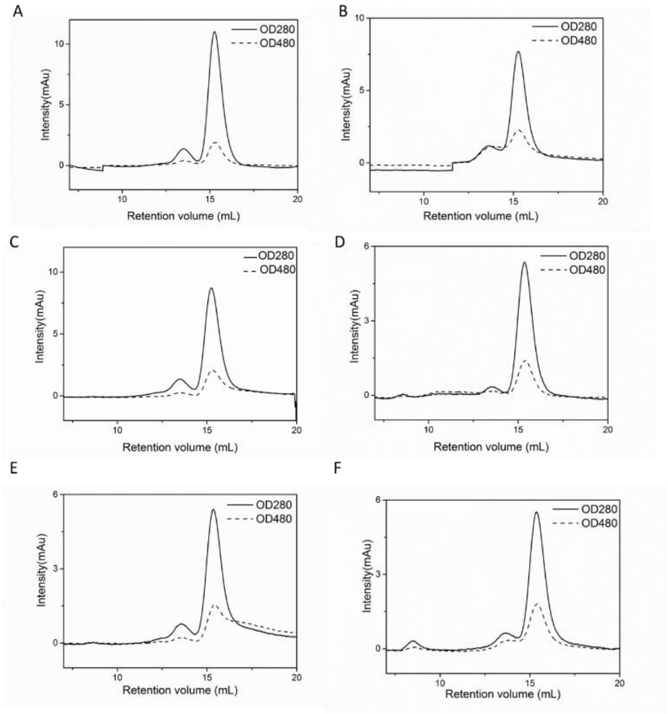

(1) Chromatograms of 45 ºC HFn/DOX samples. pH 7.0, 2 h (**A**), pH 7.5 2 h (**B**), pH 7.0, 4 h (**C**), pH 7.5 4 h (**D**), pH 7.0 6 h (**E**), pH 7.5 6 h (**F**).

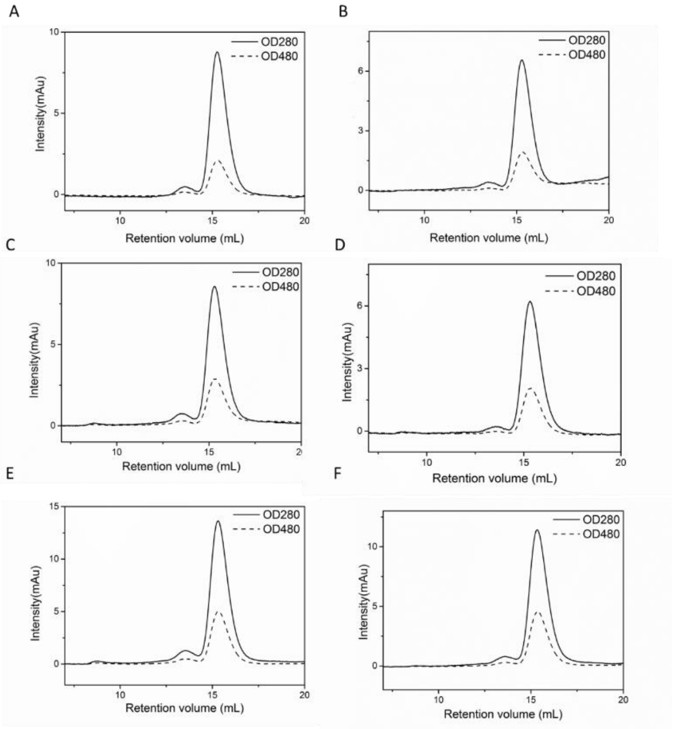

(2) Chromatograms of 50 ºC HFn/DOX samples. pH 7.0, 2 h (**A**), pH 7.5 2 h (**B**), pH 7.0, 4 h (**C**), pH 7.5 4 h (**D**), pH 7.0 6 h (**E**), pH 7.5 6 h (**F**).

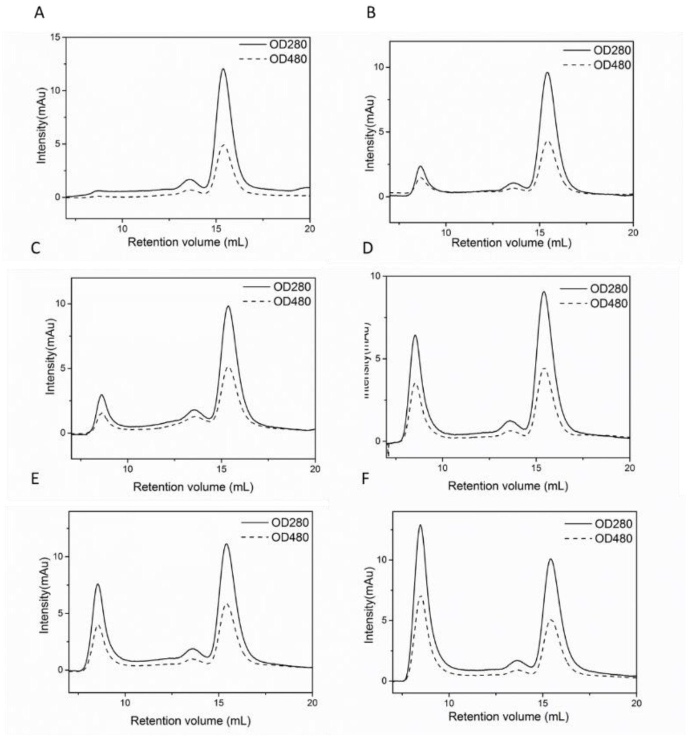

(3) Chromatograms of 60 ºC HFn/DOX samples. pH 7.0, 2 h (**A**), pH 7.5 2 h (**B**), pH 7.0, 4 h (**C**), pH 7.5 4 h (**D**), pH 7.0 6 h (**E**), pH 7.5 6 h (**F**).

Figure S2. Size exclusion chromatograms of all HFn/DOX samples under 18 conditions in thermally induced drug loading optimization.

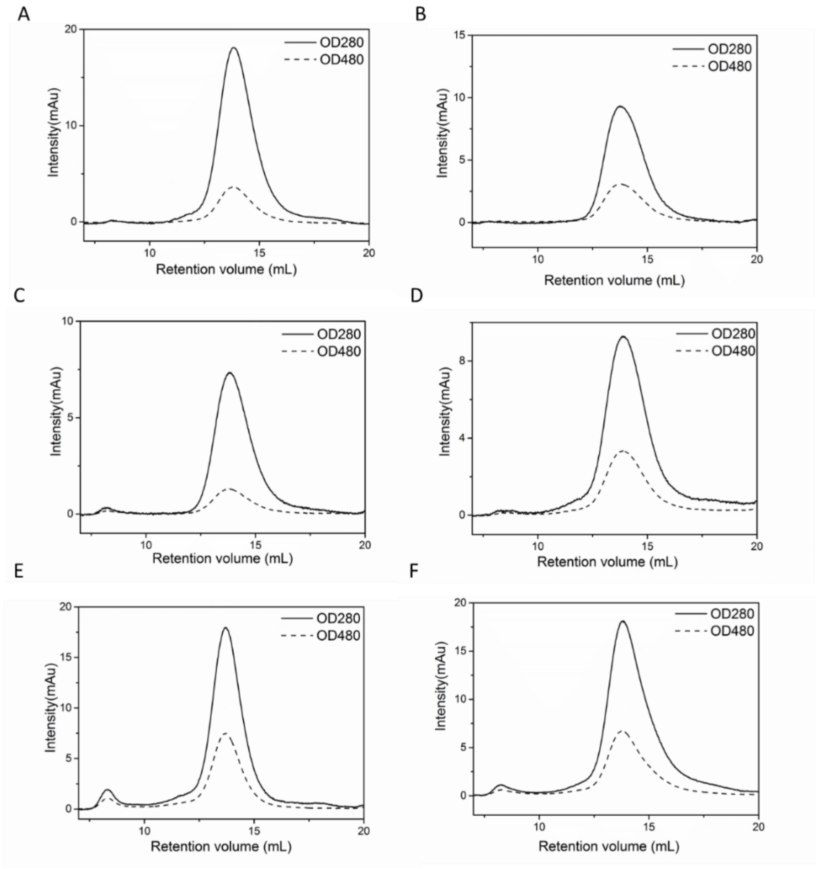

(1) Chromatograms of 45 ºC HFn-GFLG-PAS-RGDK/DOX samples. pH 7.0, 2 h (**A**), pH 7.5 2 h (**B**), pH 7.0, 4 h (**C**), pH 7.5 4 h (**D**), pH 7.0 6 h (**E**), pH 7.5 6 h (**F**).

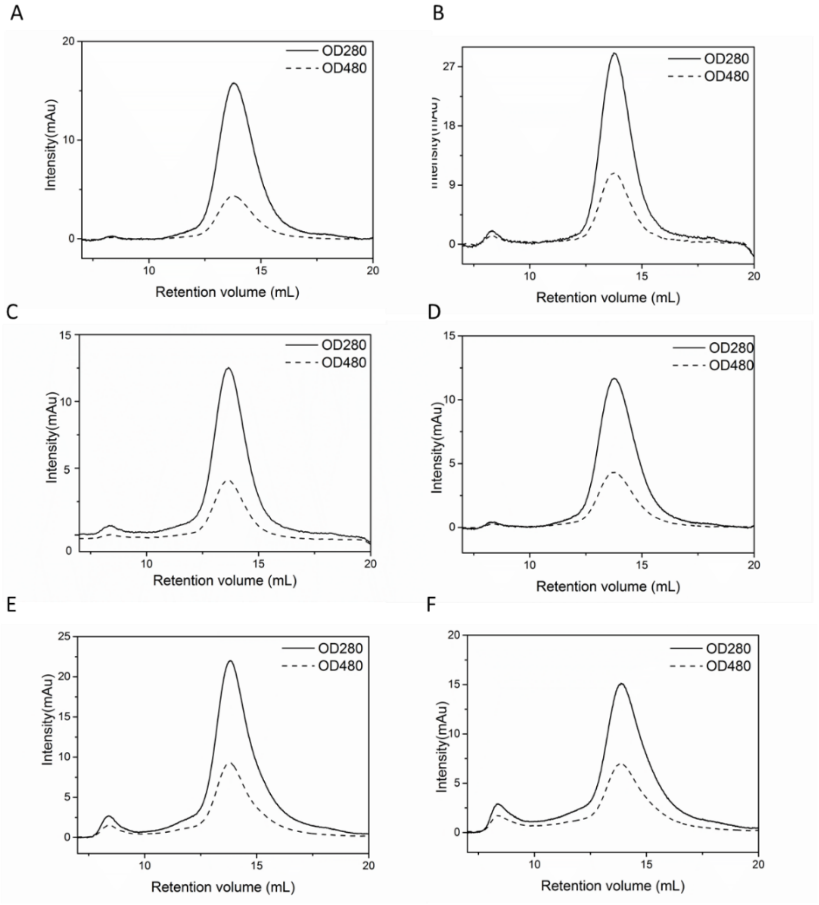

(2) Chromatograms of 50 ºC HFn-GFLG-PAS-RGDK/DOX samples. pH 7.0, 2 h (**A**), pH 7.5 2 h (**B**), pH 7.0, 4 h (**C**), pH 7.5 4 h (**D**), pH 7.0 6 h (**E**), pH 7.5 6 h (**F**).

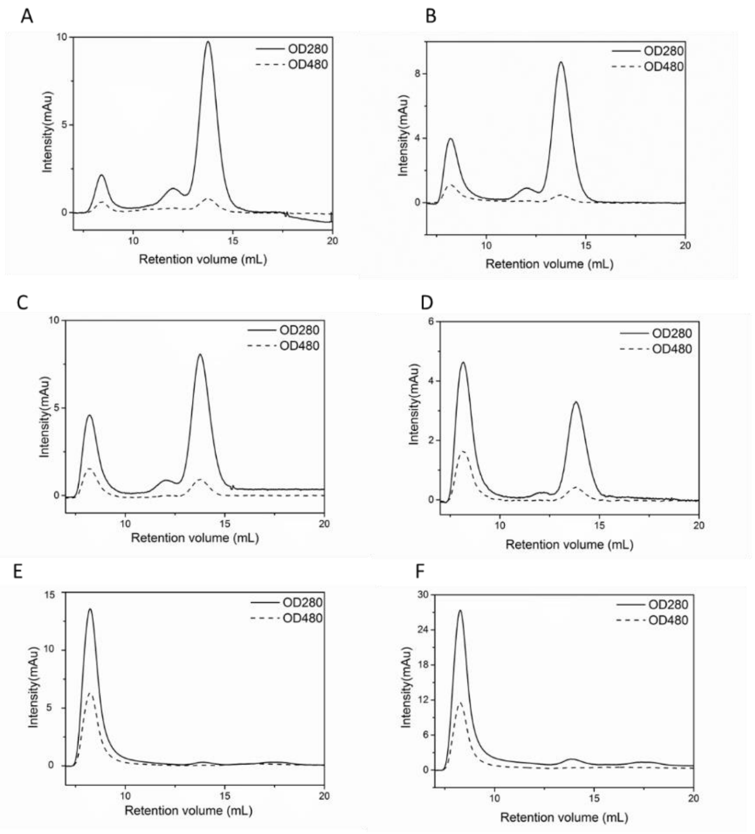

(3) Chromatograms of 60 ºC HFn-GFLG-PAS-RGDK/DOX samples. pH 7.0, 2 h (**A**), pH 7.5 2 h (**B**), pH 7.0, 4 h (**C**), pH 7.5 4 h (**D**), pH 7.0 6 h (**E**), pH 7.5 6 h (**F**).

Figure S3. Size exclusion chromatograms of all HFn-GFLG-PAS-RGDK/DOX samples under 18 conditions in thermally induced drug loading optimization.

(1) Complex 1

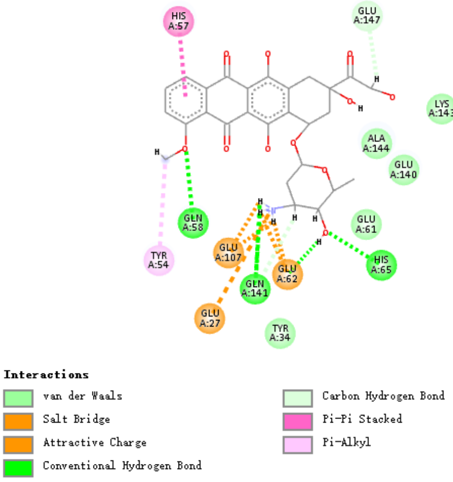

(2) Complex 4

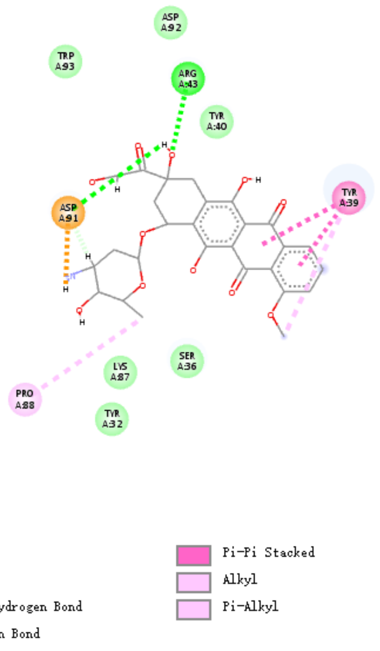

(3) Complex 5

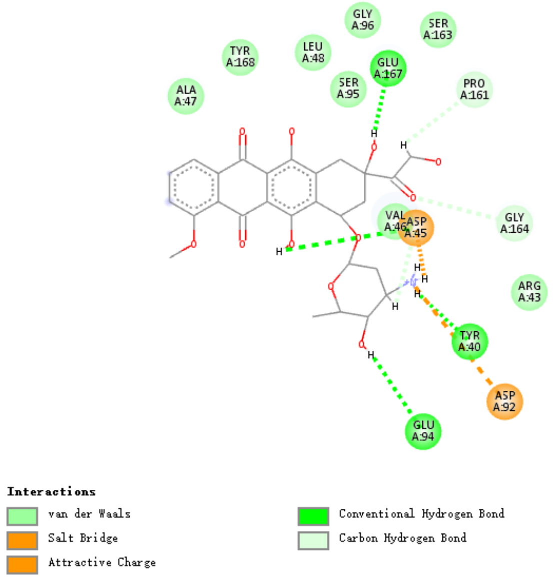

(4) Complex 3

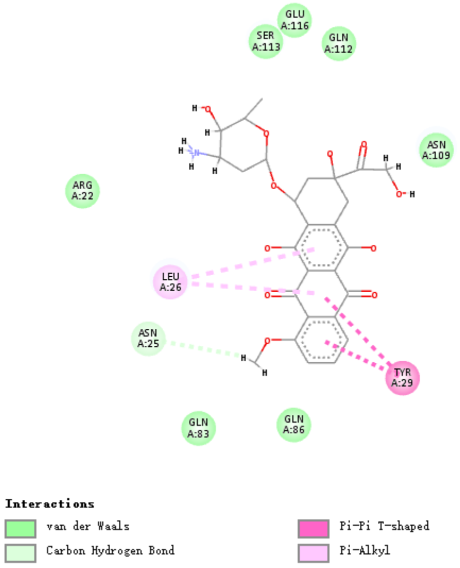

(5) Complex 2

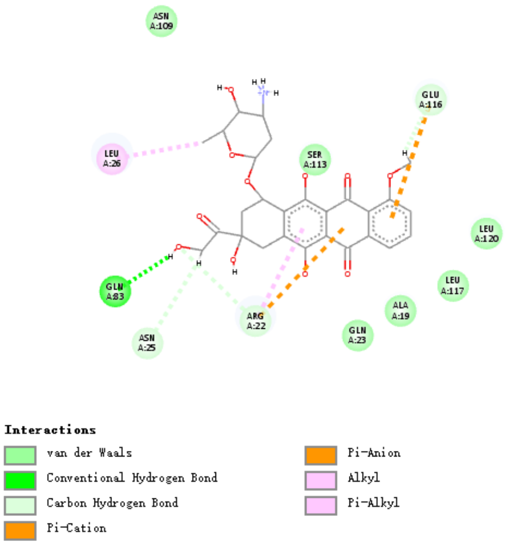

(6) Complex 6

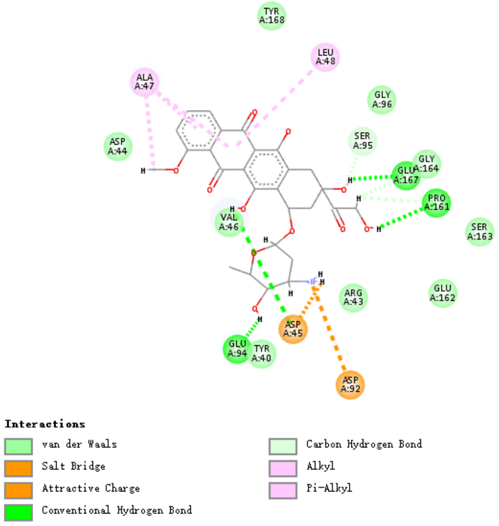

(7) Complex 7

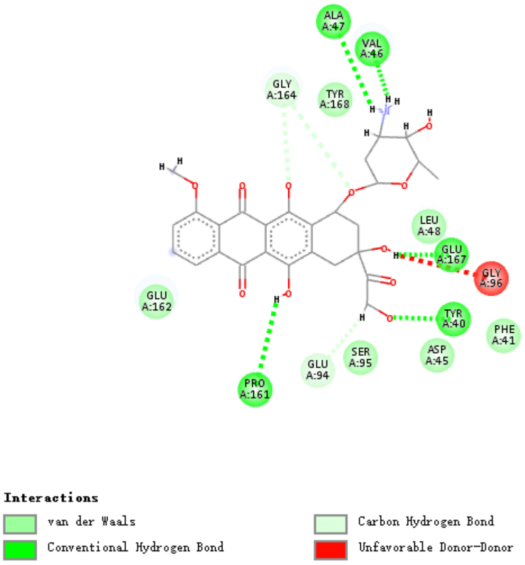

(8) Complex 8

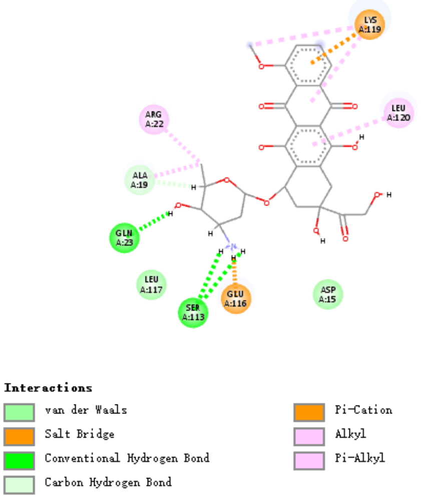

(9) Complex 9

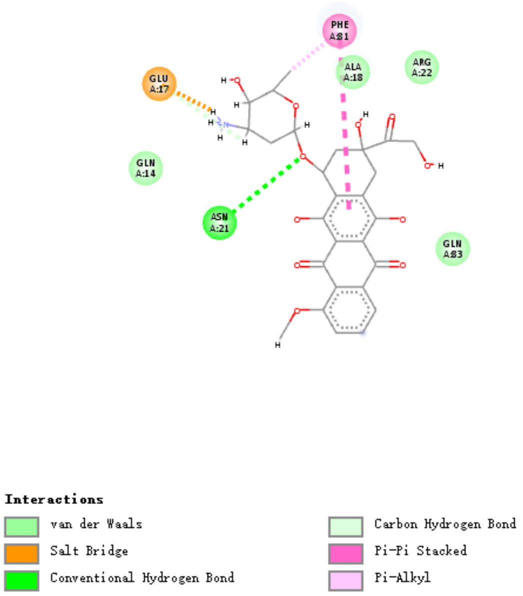

Figure S4. Hydrogen bond, salt bridge and Pi effect interactions between HFn subunit and DOX in Complex 1-9 after 10 ns MD simulation.
